# Early Inflammatory Markers for the Diagnosis of Late-Onset Sepsis in Neonates: The Nosodiag Study

**DOI:** 10.3389/fped.2018.00346

**Published:** 2018-11-13

**Authors:** Laurence Dillenseger, Claire Langlet, Silvia Iacobelli, Thomas Lavaux, Charline Ratomponirina, Marc Labenne, Dominique Astruc, François Severac, Jean Bernard Gouyon, Pierre Kuhn

**Affiliations:** ^1^Service de Pédiatrie II, Hôpital de Hautepierre, Strasbourg, France; ^2^Service de Réanimation Néonatale et Pédiatrique, Néonatologie, CHU La Réunion, Saint Pierre, France; ^3^Laboratoire de Biochimie et de Biologie Moléculaire, Hôpital de Hautepierre, Strasbourg, France; ^4^Service de Réanimation Pédiatrique, Hôpital Timone 2, Marseille, France; ^5^Service de Santé Publique, Nouvel Hôpital Civil, Strasbourg, France

**Keywords:** Late-onset neonatal sepsis, newborn infant, C-reactive protein, Procalcitonin, Interleukin 6, Interleukin 8

## Abstract

**Background:** Early diagnosis is essential to improve the treatment and prognosis of newborn infants with nosocomial bacterial infections. Although cytokines and procalcitonin (PCT) have been evaluated as early inflammatory markers, their diagnostic properties have rarely been compared.

**Objectives:** This study evaluated and compared the ability of individual inflammatory markers available for clinician (PCT, semi-quantitative determination of IL-8) and of combinations of markers (CRP_i_ plus IL-6 or quantitative or semi-quantitative determination of IL-8) to diagnose bacterial nosocomial infections in neonates.

**Methods:** This prospective two-center study included neonates suspected of nosocomial infections from September 2008 to January 2012. Inflammatory markers were measured initially upon suspicion of nosocomial infection, and CRP was again measured 12–24 h later. Newborns were retrospectively classified into two groups: those who were infected (certainly or probably) and uninfected (certainly or probably).

**Results:** The study included 130 infants of median gestational age 28 weeks (range, 24–41 weeks). Of these, 34 were classified as infected and 96 as uninfected. The sensitivity, specificity, positive and negative predictive values (PPV and NPV), and positive and negative likelihood ratios (LR+ and LR-) for PCT were 59.3% (95% confidence interval [CI], 38.8–77.6%), 78.5% (95% CI, 67.8–86.9%), 48.5% (95% CI, 30.8–66.5%), 84.9% (95% CI, 74.6–92.2%), 2.7 (95% CI, 1.6–4.9), and 0.5 (95% CI, 0.3–0.8), respectively. Semi-quantitative IL-8 had the highest specificity (92.19%; 95% CI, 82.70–97.41%), PPV (72.22%; 95% CI, 46.52–90.30%) and LR+ (6.17, 95% CI, 2.67–28.44), but had low specificity (48.15%; 95% CI, 28.67–68.05%). Of all markers tested, the combination of IL-6 and CRP_i_ had the highest sensitivity (78.12%; 95% CI, 60.03–90.72%), NPV (91.3%; 95% CI, 82.38–96.32%) and LR- (0.29; 95% CI, 0.12–0.49). The combination of IL-6 and CRP_i_ had a higher area under the curve than PCT, but with borderline significance (*p* = 0.055).

**Conclusions:** The combination of IL-6 and CRP_i_ was superior to other methods, including PCT, for the early diagnosis of nosocomial infection in neonates, but was not sufficient for sole use. The semi-quantitative determination of IL-8 had good diagnostic properties but its sensitivity was too low for use in clinical practice.

## Introduction

Nosocomial bacterial infection (NBI) increases mortality and morbidity in neonates, especially in very low birth weight or extremely preterm newborn infants. NBI-associated complications have been observed in one-quarter of these infants, lengthening their hospital stay. These infections have been associated with poor neurodevelopment and growth and with altered lung development (1, 2). A rapid diagnosis of NBI is difficult because of the low sensitivity and the lack of specificity of clinical signs as well as the delayed increase in C-reactive protein (CRP) levels and the time required to obtain full bacteriological results. Due to the lack of a perfect gold standard, the best references for the diagnosis of NBI are positive bacteriological results. However, blood cultures have inherent limitations. The number of blood samples that can be safely obtained is limited. The quality of these samples and the amount of blood available for cultures are also challenging. Insufficient sampling can yield false negative results, whereas sample contamination can yield false positive results. Owing to the possible severity of NBI, antibiotic treatment is frequently initiated immediately in neonates with a suspected diagnosis of NBI. This strategy, however, results in the exposure of a large number of newborn infants to needless antibiotic administration, carries a potential risk for the selection in these patients of multiple drug-resistant bacteria and increases health care costs. Several cytokines increasing early during inflammatory cascades, including interleukin IL-6, IL-8 and procalcitonin (PCT) have been evaluated as early inflammatory markers of NBI in neonates. Although studies have shown that these proteins have diagnostic value, they have often led to conflicting results (3–5). Recommendations of the Evidence Based Medicine Working Group (6) have attempted to standardize the methodology of studies evaluating diagnostic markers. These include the need for blinded comparisons; the inclusion in the study population of patients to whom these tests are applicable in clinical practice; and the reporting of the diagnostic properties of these markers as likelihood ratios to assess at best their clinical value.

Studies have also assessed the diagnostic properties of combinations of CRP with IL-6 and IL-8 (7–9), with these combinations clinically used in some neonatal care units. These assays required a minimum time of 85 min to obtain the results, an amount of time compatible with clinical decision making but which should be shortened. Several studies have evaluated the ability of PCT to diagnose NBI in neonates (5, 10–12), with the minimum time required by the Kryptor™ (Brahms™) assay of only 40 min. A semi-quantitative method of measuring IL-8 (Quickline™; Milenia™) has been reported to detect minimum concentrations beyond the threshold of 50 ng.L^−1^, with the results available in 20 min using a densitometer and available at the bedside. Although, all of these methods are faster and/or less expensive than the commonly used assays, their diagnostic properties have rarely been compared.

The main objectives of this study were to evaluate and compare the ability of individual inflammatory markers (PCT, CRP, quantitative and semi-quantitative determination of IL-8) and of combinations of markers (CRP plus IL-6 or quantitative or semi-quantitative determination of IL-8) to diagnose NBI in neonates.

## Materials and methods

This prospective two-center study was undertaken in the neonatal intensive care units (NICU) of the University Hospitals of Strasbourg and Dijon between September 2008 and January 2012. This trial was registered in June 2008 on the U.S. National Institutes of Health ClinicalTrials.gov website under identification number NCT00701948.

### Study population

The study included all infants aged >72 h hospitalized in the NICU with a clinical suspicion of nosocomial sepsis, based on the presence of at least three clinical criteria and at least one risk factor reported in Table 1 (13).

**Table 1:** Criteria and clinical risk factors for nosocomial bacterial infection.

**Systems**	**Clinical signs and risk factors**
Respiratory	- Tachypnea - Dyspnea - Increased need of ventilatory supports or oxygen requirements - Apnea
Hemodynamics	- Gray color - Paleness or CRT>3s - Tachycardia (HR>180 beats / min) - Bradycardia (HR <100 beats / min) - High blood pressure, or need for inotropic therapy
Digestive	- Vomiting - Abdominal distension - Increased gastric residuals - Hepatomegaly
Neurological	- Lethargy - Hypotonia or hypertonia - Irritability
Thermoregulation	- Hypothermia - Hyperthermia
Metabolic - biological -	- Hyperglycemia - Metabolic acidosis - Leukopenia (<5,000 cells /mm^3^) or leukocytosis (> 20,000 cells/mm^3^) - Thrombocytopenia
Risk factors	- Endotracheal tube - Central venous catheter - Parenteral nutrition - Presence of a nasogastric tube - Presence of a urinary catheter - Presence of ventricular derivation catheters - Postnatal corticosteroids - Pre-surgery

Newborn infants were excluded if they were in early postoperative phase (< 48 h after surgery), had major congenital anomalies, had necrotizing enterocolitis with radiological signs, had previously been included in this study for an earlier episode of suspected NBI, or had been treated with antibiotics during the 24 h prior to being suspected of NBI.

### Timing and methods of determination

Inflammatory markers were measured initially upon suspicion of nosocomial infection. Semi-quantitative IL-8 and PCT were measured at the time of measurement of IL-6 and/or quantitative IL-8 and initial CRP (CRP_i_). assays usually performed in patients with suspected infection at each center. The total volume of blood required was 1.6 ml (1.1 ml for semi-quantitative IL-8 and PCT and 0.5 ml for CRP_i_ and IL-6). If the volume of blood required for semi-quantitative IL-8 and PCT measurements was insufficient, no additional puncture was performed. An additional sample for measurement of CRP was taken from each patient 12–24 h after the first sample. The times required to obtain results were 20 min for CRP (RxL^TM^; Dade Behring^TM^), 85 min for quantitative IL-8 (Immulite^TM^; DPC^TM^), 85 min for IL-6 (Immulite^TM^; DPC^TM^), 40 min for PCT (Kryptor^TM^; Brahms^TM^), and 20 min for semi-quantitative IL-8 (Quickline^TM^; Milenia^TM^).

### Microbiological analyses

Blood samples (1 ml) for culture were systematically collected before the initiation of any treatment. Samples were incubated for 72 h, based on recommendations regarding the time to positivity of neonatal cultures (14). Cultures of other samples were based on the clinical signs exhibited by the newborn infant. Cerebrospinal fluid samples were obtained by lumbar puncture of newborns with neurological signs or blood cultures positive for particular pathogens. Tracheal aspirates were obtained through sterile maneuvers. Urine was obtained non-invasively using urine collection pads after rigorous disinfection of the perineal area or invasively using a catheter if a bladder sondage was present.

### Antibiotic treatment

The decision to start antibiotic treatment was made by the clinician in charge of each infant according to the standard protocol of each unit and based on a combination of clinical signs and inflammatory markers results. Those with life-threatening conditions or with hemodynamic clinical signs were started on antibiotics immediately. Those with moderate clinical signs were started on antibiotics only in case of elevated inflammatory markers (IL-6 or CRP_i_ in center A and quantitative IL-8 or CRP_i_ in center B) or in case of persistence. The choice of antibiotics was specific to each center. Treatments were started prior to knowledge of the semi-quantitative IL-8 and PCT assays.

### Classification

Newborn infants were retrospectively classified into two groups—infected infants (certainly or probably) and uninfected infants (certainly or probably)—by independent physicians who did not participate in the care of the children and who had no knowledge of the results of the marker assays, except for maximum CRP concentration during the episode. Infants were classified based on bacteriological, clinical, and biological criteria.

A newborn with bacteriologically proven sepsis was considered certainly infected. Septicemia was defined by the presence of a positive blood culture. Newborns were considered certainly infected with typical skin contaminants, including such as *coagulase-negative staphylococci, bacilli, propionibacteria* and yeast, if they had positive blood cultures with clinical signs of infection and a CRP concentration >10 mg/l within 12–60 h after the initial assessment of infection (7–9, 13). Meningitis was defined as positive results on lumbar puncture (>10 cells/mL). Pneumonia required the presence of pathogenic bacteria in bronchoalveolar lavage fluid (>10^4^ bacteria/mL) or in protected tracheal aspiration, with chest radiographs showing new or progressing infiltrates, worsening of gas exchange or an increase in the requirements of an intubated infant for ventilator support, and at least four of the following symptoms: fever or hypothermia, apnea/bradycardia, tachypnea, increased secretions, purulent secretions, increased CRP levels, and neutrophilia. Pyelonephritis was defined as the co-occurrence of the clinical signs of sepsis and CRP levels >10 mg/l within 12–60 h after the initial assessment of infection and by the presence in urine of >10^6^ cells/l and >10^5^ bacteria/ml.

Newborns were considered probably infected if they had clinical signs of infection, CRP ≥ 10 mg/l within 12–60 h after the initial assessment of infection, had no alternative diagnosis, and showed improvement in clinical status following treatment with antibiotics.

Newborns were considered probably not infected if an alternative diagnosis could explain their clinical signs or elevated CRP, if stool cultures or tracheal aspirates were positive for bacteria in the absence of clinical or biological signs of infection, if their blood culture was positive but CRP was < 4 mg.L^−1^ or if they showed favorable outcomes following antibiotic treatment for <5 days.

Newborns were classified as certainly not infected if they showed clinical improvement and a normalization of CRP levels without antibiotics.

### Statistical analysis

Normally distributed quantitative values were expressed as means and standard deviations and compared using Fisher's *t*-tests, whereas non-normally distributed values were expressed as medians and ranges and compared using Mann-Whitney U-tests.

Diagnostic properties were analyzed using Bayes' theorem, with the results expressed as sensitivity, specificity, negative predictive value (NPV), positive predictive value (PPV), positive likelihood ratio (LR+), and negative likelihood ratio (LR-). Receiver operating characteristic (ROC) curve analyses of combinations of markers were performed using binary criteria: if one of the two levels exceeded its threshold value, the result was considered positive. Areas under the curve were compared using the DeLong test (15). In all statistical analyses, a *P* value < 0.05 was considered statistically significant.

### Ethics

The study protocol was approved by the institutional review board of each hospital and by the ethics committee East IV in June 2008 (approval number 08/30). Both parents of each newborn provided written informed consent.

## Results

A total of 130 infants were included in the study. There were 61 girls and 69 boys. The median gestational age was 28 weeks (24-41) and median birth weight was 1,037 grams (580–3,880). The characteristics of the study population are presented in Table 2. Of the 130 patients, 34 (26.2%) were classified as infected, including 18 certainly infected and 14 probably infected; and 96 (73.8%) were classified as not infected, including 65 certainly not infected and 31 probably not infected. The most frequently detected bacterium was methicillin resistant coagulase negative *Staphylococcus*. Seventeen children had septicemia, and one had pyelonephritis.The pathogens identified in the positive blood cultures are presented in Table 3. No child in this study died during the infectious episode.

**Table 2:** Characteristics of the study population.

	**Infected *n* = 34**	**Not infected *n* = 96**	***p***
Male/Female	15/18	46/51	0.84
Gestational age (wk) Median (range)	27 (24–41)	28 (24–41)	0.06
Birthweight (g) Median (range)	888 (604–3,300)	1070 (580–3,880)	0.03
Duration of antibiotic treatment (d) Median (range)	9 (2–20)	0 (0–9)	0.0001
Postnatal age at onset of infection (d) Median (range)	11 (4–109)	12 (4–58)	0.83
CRIB score Mean ±*SD*	10.9 ± 3.8	8.8 ± 3.5	0.006

**Table 3:** List of pathogens identified in positive blood cultures.

**Bacteria**	**Methicillin-sensitive Coagulase Negative Staphylococci**	**Methicillin-resistant Coagulase Negative Staphylococci**	***Bacillus cereus***	***Pseudomonas aeruginosa***	***Staphylococcus capitis***
Number of cases	3	9	1	1	3

The median time between the onset of clinical signs and blood sampling was 4.7 h (0–32 h).

### Diagnostic properties of inflammatory markers

Table 4 shows the diagnostic properties of the different markers and their combinations. The optimal threshold values were obtained using the ROC curve methods. Of all markers tested, both alone and combined, the combination of IL-6 and CRP_i_ has the highest sensitivity (78.12%; 95% confidence interval [CI] 60.03–90.72%), NPV (91.3%; 95% CI 82.38–96.32%) and LR- (0.29; 95% CI 0.12–0.49). Semi-quantitative measurement of IL-8 had the highest specificity (92.19%; 95% CI 82.70–97.41%), PPV (72.22%; 95% CI 46.52–90.30%), and LR+ (6.17; 95% CI 2.67–28.44) but had a low sensitivity (48.15%; 95% CI 28.67–68.05%).

**Table 4:** Diagnostic properties of the different inflammatory markers.

**Test**	**Threshold values**	**Se (%)**	**Sp (%)**	**PPV (%)**	**NPV (%)**	**LR+**	**LR-**
CRP_i_ *n* = 130	4.05 mg/l	70.59	84.21	61.54	88.89	4.47	0.35
		(52.52–84.90)	(75.30–90.88)	(44.62–76.64)	(80.51–94.54)	(2.79–8.38)	(0.17–0.54)
Quantitative IL-8 *n* = 114	107 ng/l	40,62	86.59	54.17	78.89	3.03	0.69
		(23.70–59.36)	(77.26–93.11)	(32.82–74.44)	(69.01–86.79)	(1.54–6.66)	(0.48–0.89)
Semi-quantitative IL-8 *n* = 91	77.5 ng/l	48.15	92.19	72.22	80.82	6.17	0.56
		(28.67–68.05)	(82.70–97.41)	(46.52–90.30)	(69.92–89.10)	(2.67–28.44)	(0.36–0.78)
PCT *n* = 106	0,69 μg/l	59.26	78.48	48.48	84.93	2.75	0.52
		(38.80–77.61)	(67.80–86.94)	(30.80–66.46)	(74.6–92.23)	(1.61–4.95)	(0.29–0.78)
IL-6/CRP_i_ *n* = 126	21.7 ng/l−4.05 mg/l	78.12	76.34	53.19	91,03	3.30	0.29
		(60.03–90.72)	(66.40–84.54)	(38.08–67.89)	(82.38–96.32)	(2.25–5.27)	(0.12–0.49)
Quantitative IL-8 / CRP_i_ *n* = 114	107 ng/l−4.05 mg/l	75.00	75.31	54.55	88,41	3.04	0.33
		(56.60–88.54)	(64.47–84.22)	(38.85–69.61)	(78.43–94.86)	(2.01–5.06)	(0.13–0.55)
Semi–quantitative IL-8 / CRP_i_ *n* = 91	77.5 ng/l−4.05 mg/l	74.07	77.78	58,82	87,50	3.33	0.33
		(53.72–88.89)	(65.54–87.28)	(40.70–75.35)	(75.93–94.82)	(2.09–6.13)	(0.13–0.57)

The values in brackets indicate the 95% confidence intervals.

*Se, Sensitivity ; Sp, Specificity; PPV, Positive predictive values; NPV, Negative predictive values; LR+, Positive likelihood ratios; LR-, Negative likelihood ratios*.

In Table 5, we present the diagnostic properties of the different markers and their combination calculated only in certainly infected infants and after exclusion of probably infected ones.

**Table 5:** Diagnostic properties of the different inflammatory markers in the certainly infected infants.

**Test**	**Threshold values**	**Se (%)**	**Sp (%)**	**PPV (%)**	**NPV (%)**	**LR+**	**LR-**
CRP_i_ *n* = 83	7.82 mg/l	66.67	89.23	63.16	90.62	6.19	0.37
		(40.99; 86.66)	(79.06; 95.56)	(38.36; 83.71)	(80.70; 96.48)	(2.86; 13.40)	(0.19; 0.72)
Quantitative IL-8 *n* = 69	74.13 ng/l	43.75	89.09	53.85	84.48	4.01	0.63
		(19.75; 70.12)	(77.75; 95.89)	(25.13; 80.78)	(72.58; 92.65)	(1.57; 10.24)	(0.41; 0.98)
Semi quantitative IL-8 *n* = 52	77.72 ng/l	53.85	95.24	77.78	86.96	11.31	0.48
		(25.13; 80.78)	(83.84; 99.42)	(39.99; 97.19)	(73.74; 95.06)	(2.67; 47.88)	(0.27; 0.88)
PCT *n* = 63	1.5 μg/l	53.85	98.04	87.5	89.29	27.46	0.47
		(25.13; 80.78)	(89.55; 99.95)	(47.35; 99.68)	(78.12; 95.97)	(3.70; 203.91)	(0.26; 0.85)
IL-6/CRP_i_ *n* = 79	21.4–7.82 mg/l	75	84.13	54.55	92.98	4.72	0.3
		(47.62; 92.73)	(72.74; 92.12)	(32.21; 75.61)	(83.00; 98.05)	(2.50; 8.92)	(0.13; 0.70)
Semi quantitative IL-8/CRP_i_ *n* = 52	77.72 ng/l−7.82 mg/l	69.23	88.1	64.29	90.24	5.82	0.35
		(38.57; 90.91)	(74.37; 96.02)	(35.14; 87.24)	(76.87; 97.28)	(2.37; 14.29)	(0.15; 0.80)

### Comparison of the areas under the curve

**Figure 1:**
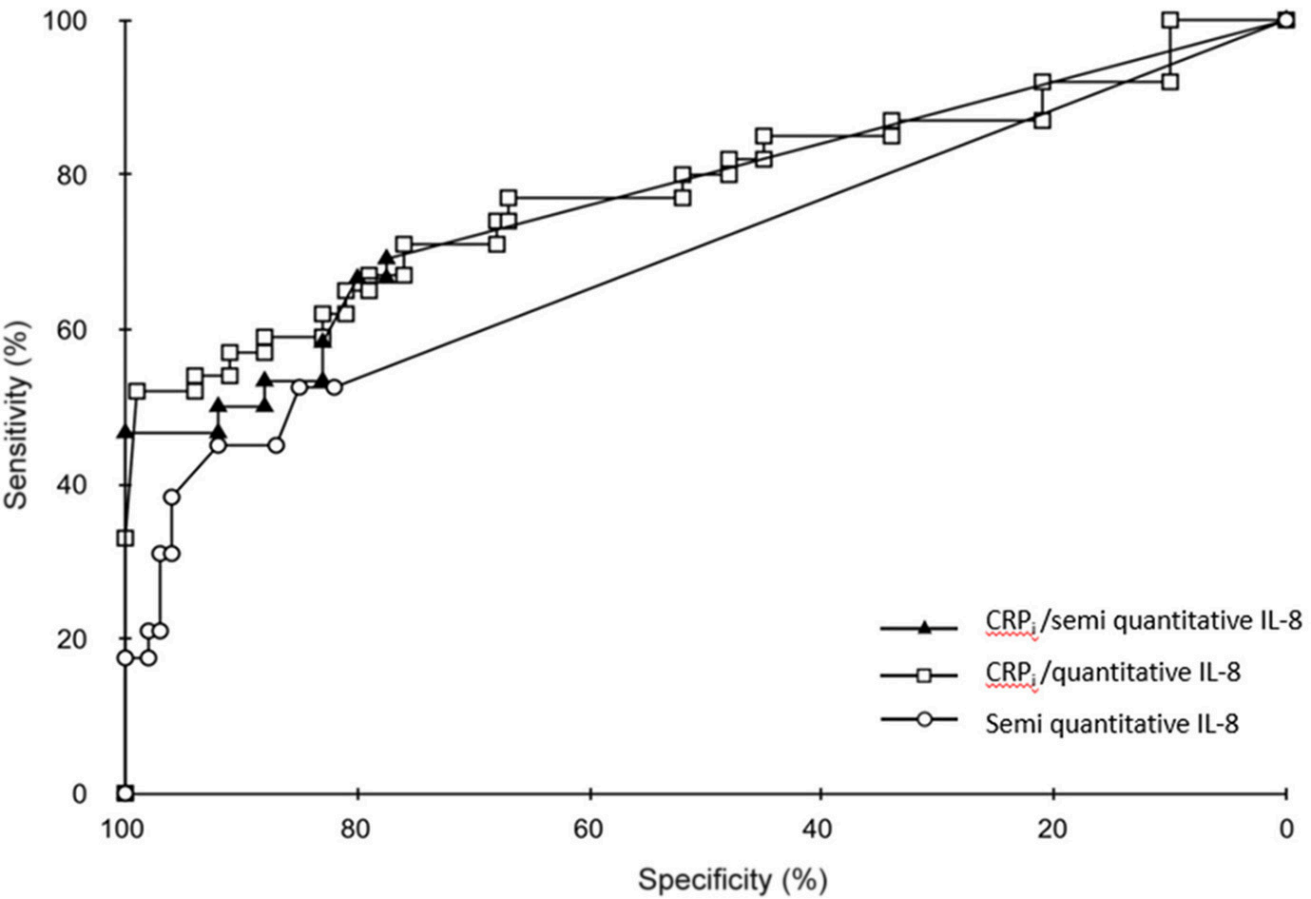
Receiver operating characteristic (ROC) curves for CRPi plus semi quantitative determination of IL-8, CRPi plus quantitative determination of IL-8 and semi-quantitative determination of IL-8 alone.

**Figure 2:**
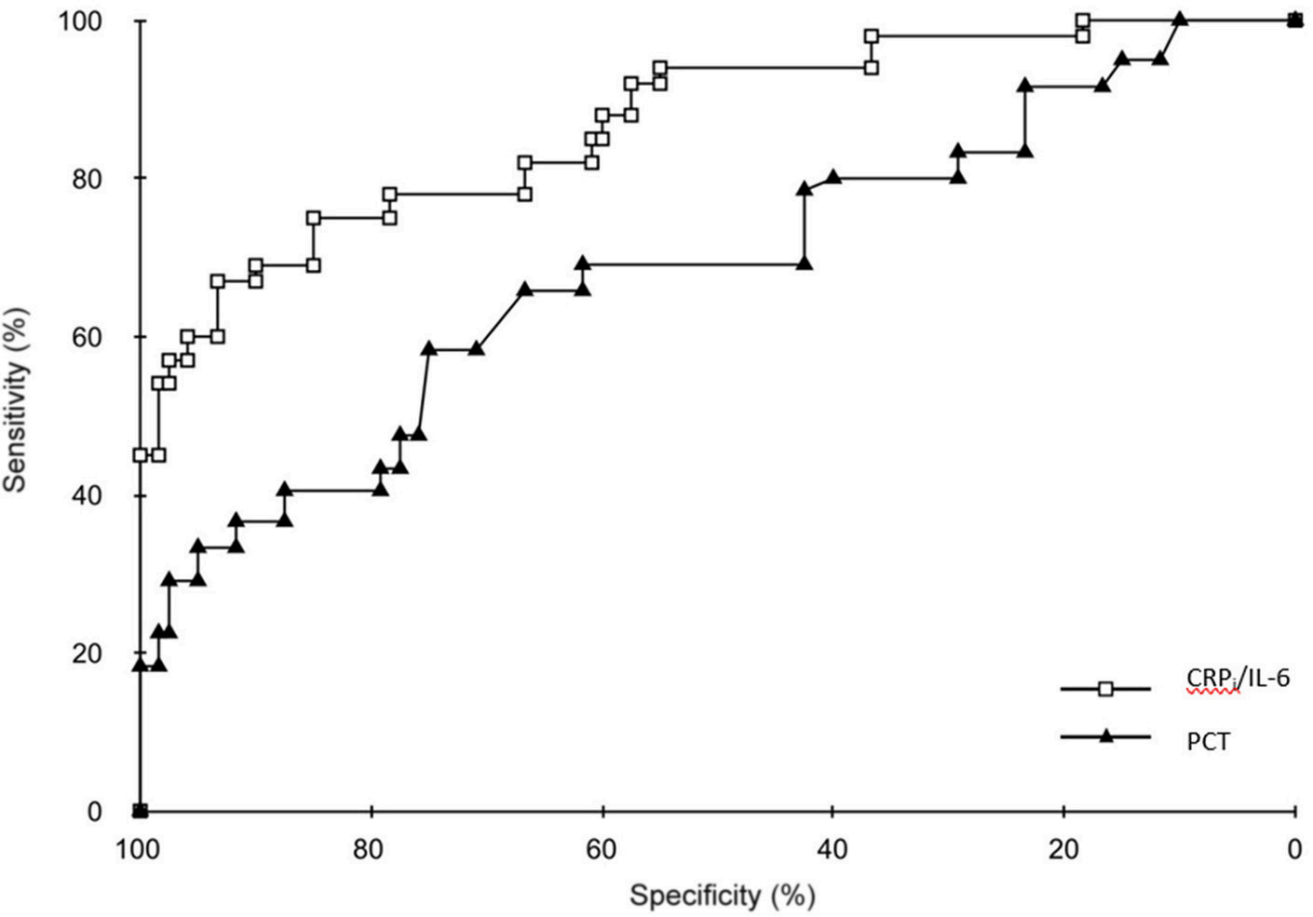
Receiver operating characteristic (ROC) curves for CRP_i_ plus determination of IL-6, PCT alone.

ROC curves for each of the markers or combination of markers are shown in Figures 1, 2. There were no significant differences between the AUCs of PCT and the combinations of CRP_i_ with quantitative and semi-quantitative measurement of IL-8. However, the AUC for the combination of IL-6 and CRP_i_ tended to be significantly superior to the AUC for PCT (*p* = 0.055; Table 6).

**Table 6:** Comparison of areas under the curves for the different marker.

**Markers**	**AUC**	**CI 95%**	P^*^
PCT vs. IL-6/CRP_i_	71.65–84.80	(57.74–85.56)–(75.03–96.58)	0.055
PCT vs. quantitative IL-8/CRP_i_	71.65–80.51	(57.74–85.56)–(67.51–93.51)	0.330
PCT vs. Semi-quantitative IL-8/CRP_i_	71.65–81.44	(57.74–85.56)–(69.88–93.00)	0.253
Semi-quantitative IL-8/CRP_i_ vs. IL-6/CRP_i_	81.44–85.80	(69.88–93.00)–(75.03–96.58)	0.336
Quantitative IL-8/CRP_i_ vs. IL-6/CRP_i_	80.51–85.80	(67.51–93.51)–(75.03–96.58)	0.288

* *Delong test*.

## Discussion

This study compared the performance of currently available inflammatory markers to diagnose NBI in all newborn infants suspected of NBI. The optimal assay should be rapid, available round the clock, and inexpensive. It should be reliable and reproducible with excellent diagnostic properties, including a clearly determined threshold value for the diagnosis of NBI. Moreover, because it is used for diagnosis in newborns, these assays should require as little blood as possible and identify all infected infants immediately after infection is suspected. Moreover, the real value of a diagnostic test should be determined by calculating likelihood ratios (16).

This study assessed two markers that can be measured separately and combination of markers. Semi-quantitative assays of IL8 have the potential advantages of rapidity and use at the bedside. This marker showed high specificity, with good LR+ and LR- close to the recommended optimal values of >10 and < 0.1, respectively (16). However, the best threshold value determined in our study was higher than the one recommended by the developer of the technique (50 μg/l). Moreover, this test had relatively low sensitivity, < 50%, limiting its usefulness. PCT had acceptable specificity, but low sensitivity, around 60%, and far from ideal LR + and LR-, findings similar to those observed in several studies (7, 12, 17). Although other studies have reported that PCT has better diagnostic properties for NBI (5, 10, 11), those studies included control groups consisting of healthy newborns with no suspicion of infection, a methodology found to artificially increase the diagnostic accuracy of the tested markers (6). Other studies, however, have reported that PCT is a better marker for NBI than CRP, with PCT found to have a greater sensitivity and a lower LR- than CRP during initial stages of infection (18–20). Another study reported also that this assay had a better global diagnostic performance (21). Thus, our finding of better diagnostic properties of CRP as compared to PCT alone is intriguing. It could be explained by several factors. First, a delayed sampling in the course of infection, possibly suggested by the time elapsed between the onset of clinical signs determined retrospectively and the timing of sampling, could explain the better performances of CRP, which rises in the circulation 8–12 h after the onset of infection. Second, the inclusion of probably infected infants in the whole analysis, has certainly increased the diagnostic properties of CRP. This hypothesis is partly supported by the fact that the diagnostic properties of PCT are a bit better than the ones of CRP when calculated only in infants with proven NBI. Finally, we observed a threshold value between 0.5 and 1 μg/l, whereas a large multicenter study recommended the use of higher threshold values and that these values should vary according to the birthweight of the newborns (22). Anyway, the variability in LR and the large confidence intervals suggest the need for caution when using this single marker for the diagnosis of NBI.

Our results confirm that combinations of cytokine and CRP assays yielded a more accurate diagnosis than any individual marker. In our study, quantitative IL-8 plus CRP_i_ and IL-6 plus CRP_i_ had the best diagnostic properties. This finding appears logical based on the kinetics of inflammatory cascades. IL-6 is the main stimulator of hepatocyte synthesis of CRP, but has a short half-life, as does IL-8. The combination of these two cytokines with CRP can enable the continuous detection of inflammatory phenomena resulting from infection. The combination of IL-6 and CRP_i_ had the best sensitivity, specificity and LR-. Use of these assays in combination could reduce the number of antibiotics with an excellent NPV and good LR- while maintaining high sensitivity. This hypothesis is also supported by the strong LR- observed in other studies (3, 4, 9). The combination of IL-8 and CRP_i_ also showed excellent diagnostic performance, similar to that of IL-6 and CRP_i_ (8, 23).

Assays of other cytokines, including IL-10, TNF alpha, IL-1 beta, and IL-12 are currently available (24, 25). These techniques appear reliable, sensitive, reproducible and automatable, but are not yet used in clinical practice. The blood volumes required are very low (50–100 μL of serum), although assay times are somewhat longer (2–3 h). Lipopolysaccharide binding protein (LBP) has been reported to be a more accurate diagnostic marker than IL-6, IL-8 and PCT for the diagnosis of infections in neonates (26, 27). More recently, a new marker, presepsin, was identified; however, those studies included a small number of infants and used uninfected healthy newborn infants as a control group (28–30). Multiplex PCR assays using small volumes of blood are also being developed to detect the DNA of several bacterial species (31, 32). Although these assays were shown to be faster and more accurate than blood cultures, several hours were required to obtain their results (32).

The limitations of our study are first the relatively small number of certainly infected infants. Second, the classification into “infected” and “not infected” infants is very challenging and a possible limitation for observational studies. However, this challenge exists in all studies regarding neonatal infection. We think that it is essential to include also the group of probably infected infants in the determination of the diagnostic properties of inflammatory markers. We have to consider them as infected infants to encompass all the situations met in the daily clinical life. Finally, our study was not designed to assess the ability of markers to guide antibiotic treatment. The combination of IL-8 and CRP was found, however, to result in significant reductions in unnecessary antibiotics and cost of care, without risk to the study population (8). Similar results were found for early–onset neonatal infections, in a large multicenter randomized study involving 1291 infants admitted for suspected infections and clinically stable to wait the results of the assays (33). To our knowledge, however, no studies to date have confirmed the results of the latter study in neonates with NBI. The results of cytokine assays may be integrated into clinical and biological algorithms, similar to those proposed for early onset neonatal infection (34, 35). In these algorithms, PCT concentration in cord blood was found to be an efficient marker that could guide the treatment of newborns suspected of early onset neonatal sepsis (34–37). This approach could also include bacterial DNA load, as assessed by PCR multiplex assays (32).

## Conclusion

The combinations of CRP_i_ with IL-6 and IL-8 were superior to individual assays in the early diagnosis of NBI. The combination of IL-6 plus CRP_i_ was especially useful, but was not sufficient for sole use, and PCT was inferior to the combination of IL-6 plus CRP_i_. The semi-quantitative determination of IL-8 had good diagnostic properties but its sensitivity was too low for use in clinical practice. The benefit of this combination to rationalize antibiotic treatment should be evaluated with clinical and biological decision algorithms, while awaiting the identification of more efficient markers that are clinically usable.

## Author contributions

LD contributed to the data recording and analysis, wrote the first draft of the manuscript and edited the tables and figures. CL reviewed, the data analysis, contributed to the patient enrollment, data recording, and analysis, contributed to the writing of the first draft of the manuscript. SI, ML, JG, and DA participated in the study protocol development, enrolled patients in their department, contributed to the data recording and approved the final draft of the manuscript. TL, and CR contributed to the study protocol development, performed the markers measurement and analysis, ensured the quality of the measurements and the anonnimity of the results. FS coordinated and performed the statistical analyses, contributed to the edition of the figures and approved the final draft of the manuscript. PK coordinated the study deveploment and wrote the initial protocol, participated in the study enrollment, contributed to the writing of the manuscript and reviewed and approved the final draft.

### Conflict of interest statement

The authors declare that the research was conducted in the absence of any commercial or financial relationships that could be construed as a potential conflict of interest.
